# Reliability and validity of the Japanese version of the Community Integration Measure for community-dwelling people with schizophrenia

**DOI:** 10.1186/s13033-017-0138-2

**Published:** 2017-04-17

**Authors:** Ai Shioda, Etsuko Tadaka, Ayako Okochi

**Affiliations:** 1 Graduate School of Health Management, Keio University, 4411, Endo, Fujisawa, Kanagawa 252-0883 Japan; 2 Department of Community Health Nursing, Graduate School of Medicine, Yokohama City University, 3-9, Fukuura, Kanazawa-ku, Yokohama, 236-0004 Japan

**Keywords:** Community integration, Japanese, Reliability, Scale development, Schizophrenia, Validity

## Abstract

**Background:**

Community integration is an essential right for people with schizophrenia that affects their well-being and quality of life, but no valid instrument exists to measure it in Japan. The aim of the present study is to develop and evaluate the reliability and validity of the Japanese version of the Community Integration Measure (CIM) for people with schizophrenia.

**Methods:**

The Japanese version of the CIM was developed as a self-administered questionnaire based on the original version of the CIM, which was developed by McColl et al. This study of the Japanese CIM had a cross-sectional design. Construct validity was determined using a confirmatory factor analysis (CFA) and data from 291 community-dwelling people with schizophrenia in Japan. Internal consistency was calculated using Cronbach’s alpha. The Lubben Social Network Scale (LSNS-6), the Rosenberg Self-Esteem Scale (RSE) and the UCLA Loneliness Scale, version 3 (UCLALS) were administered to assess the criterion-related validity of the Japanese version of the CIM.

**Results:**

The participants were 263 people with schizophrenia who provided valid responses. The Cronbach’s alpha was 0.87, and CFA identified one domain with ten items that demonstrated the following values: goodness of fit index = 0.924, adjusted goodness of fit index = 0.881, comparative fit index = 0.925, and root mean square error of approximation = 0.085. The correlation coefficients were 0.43 (p < 0.001) with the LSNS-6, 0.42 (p < 0.001) with the RSE, and −0.57 (p < 0.001) with the UCLALS.

**Conclusions:**

The Japanese version of the CIM demonstrated adequate reliability and validity for assessing community integration for people with schizophrenia in Japan.

**Electronic supplementary material:**

The online version of this article (doi:10.1186/s13033-017-0138-2) contains supplementary material, which is available to authorized users.

## Background

In 2014, 773,000 people with schizophrenia were treated in Japan. Of these, 165,800 were admitted to psychiatric hospitals; people with schizophrenia accounted for 62.4% of all inpatients with mental disorders and spent an average of 546.1 days in the hospital [1]. Historically, psychiatric care in Japan has primarily been dependent on hospitalization. Community resources for psychiatric care, such as accommodations, daytime activities and daily living support services, were lacking for community-dwelling people with schizophrenia. In addition, family burdens were considerable because Japanese families believed that a family member with schizophrenia should be cared for by the family, and home confinement was the main way to keep people with schizophrenia out of the public eye. Japan still has many psychiatric beds, and remarkably, the length of hospital stay for schizophrenic patients in Japan is the longest in the world [1]. A recent study showed that the total societal cost of schizophrenia in Japan was estimated to be JPY 2.77 trillion, and this tremendous societal burden was similar to that of other developed countries [2]. Therefore, the Japanese government has decided to decrease the target number of inpatients with schizophrenia. The report titled “Toward further reform of mental health care and welfare in Japan” described a “shift in focus from the inpatient treatment of disabled people to working with these people to support and assist their integration into community life” [3]. Since this shift, it is estimated that the number of people with schizophrenia living in the community has increased.

Community integration is an essential right for people with disabilities because it promotes their physical and mental health, life satisfaction, well-being and quality of life (QOL) [4, 5]. Community integration and related concepts have been defined in various ways in the academic literature [6–13]. Many authors have described the concept as multidimensional or encompassing numerous factors. In particular, in the psychiatric and mental health fields, the dimension of environment is a necessary component. Communities in which people with disabilities feel integrated provide them with opportunities and places to learn community rules and participate in community activities, which leads to a sense of belonging [9, 12]; additionally, integrated communities provide distal support via casual community relationships through regular contact with other people who live and work in the same community [13, 14]. These concepts are consistent with the concept of community integration, as defined by McColl et al. [15]: community integration is made up of the four components of assimilation, support, occupation, and independent living.

The Community Integration Measure (CIM) was originally developed by Dr. McColl, a researcher in the field of health sciences, to assess the level of community integration among community-dwelling people with acquired brain injury [15]. There are many scales for measuring community integration in the context of individuals with psychiatric disabilities: the Social Inclusion Questionnaire User Experience (SInQUE) [16], the Social and Community Opportunities Profile (SCOPE) [17], and the Social Inclusion Scale (SIS) [18]. However, these scales have several limitations when applied in a Japanese context. First, they consist of 22–121 items and take participants a great amount of time to answer. Second, the specificity of the issue for which each scale was originally developed makes it difficult to generalize their respective results. For example, the SInQUE is based on the UK Poverty and Social Exclusion Survey. In contrast, the CIM has fewer items (ten) and a much more general content than existing instruments [16–18]; additionally, it is potentially applicable in other cultural contexts [19]. Previous studies from Canada [15], the United States [13], and Australia [4] have shown that the CIM can be applied to different cultures and ethnicities and is suitable for individuals with psychiatric disorders, including schizophrenia. However, no valid version of this instrument exists in Japan.

The aim of this study was to develop and evaluate the reliability and validity of the Japanese version of CIM—translated from the English version—for community-dwelling people with schizophrenia to enhance their community integration and promote their QOL.

## Methods

### Design

This was a cross-sectional validation study.

### Participants

The participants were community-dwelling people with schizophrenia living in the urban areas of Yokohama and Kawasaki in Kanagawa, Japan. The inclusion criteria for the participants were as follows: (1) diagnosed with schizophrenia according to the International Classification of Diseases-10; (2) living in the community; (3) aged 18–64 years; and (4) discharged for at least one and a half years from a psychiatric hospital. The participants were recruited from among the users of a local activity support center in Japan because people with schizophrenia already living in the community and those who have just been discharged from psychiatric hospitals are encouraged to use local activity support centers to reintegrate into the community [20].

The researchers sent the questionnaire and an informed consent letter to each local activity support center via mail. Each participant was asked to complete a self-administered, anonymous questionnaire voluntarily between August 1 and October 31, 2014. Before the start of the study, written informed consent for participation was obtained from the Legal Authorized Representative (LAR) guardians of the patients with schizophrenia. Moreover, informed consent was obtained from all the individual participants included in the study.

### Translation and assessment of face validity

The CIM was translated from English to Japanese by the authors and then back translated by another two English-Japanese bilingual translators. Permission was given to translate the English version into Japanese by Dr. McColl, who developed the original CIM. The face validity of the Japanese version was independently reviewed by five researchers (a professor of public health with a MD; a professor of mental health and psychiatric nursing with a PhD, a professor of health sciences and nursing with a PhD; an associate professor of community health nursing with a PhD; and an assistant professor of community health nursing with a MS) and 17 people with schizophrenia living in a community who had met the criteria for this study. These participants were not included in the main study.

### Measures

The original CIM was developed in Canada for people with traumatic brain injuries [15], though the contents are applicable to all people with disabilities who face challenges in their integration into community life [12]. McColl et al. developed a theoretical model of community integration from the perspective of people with disabilities, and the CIM’s definition of community integration focuses on the subjective experiences of integration rather than on its observable components. The CIM is based on this client-centered model [15]. The CIM consists of 10 items with no definite cut-off score for community integration. A previous study reported a mean CIM score of 37.0 (SD = 8.6) for people with schizophrenia or bipolar disorder who use public mental health services [9]. The total number of questionnaire items in the present study was 48, which addressed the following topics: demographic characteristics 12 items, social network 6 items, self-esteem 10 items, and loneliness 20 items. The Japanese version of the CIM included the following questions: “I feel like a part of this community, like I belong here;” “I can be independent in this community;” and “I have something to do in this community during the main part of my day that is useful and productive.” Responses were coded as follows: 5 = always agree, 4 = sometimes agree, 3 = neutral, 2 = sometimes disagree, and 1 = always disagree. The total CIM score ranged from 10.0 to 50.0, with higher scores indicating higher degrees of community integration. The reliability of the original CIM has been established (Cronbach’s alpha = 0.87), and its validity has been tested using a correlation with the Interpersonal Support Evaluation List.

The demographic characteristics of the participants in this study included basic characteristics such as age, sex, living status, household membership, marital status, educational status, employment status, budget, and items pertaining to features of schizophrenia, such as the cumulative number of years of psychiatric hospitalization.

Three other scales were administered to assess the concurrent validity of the Japanese version of the CIM.

Social networks were measured using the Japanese version of the Lubben Social Network Scale-Abbreviated (LSNS-6) [21]. The LSNS-6 assesses the concept of social networks, including their functions, such as social support [22]; this concept has been examined previously [15]. The scale consists of six items. Responses to the scale are assessed using a 6-point scale. The total score ranges from 0.0 to 30.0, with high scores indicating a good social network. A total score below 12.0 on the LSNS-6 identifies people at risk of social isolation. The scale’s reliability has been established with a Cronbach’s alpha of 0.82, and its validity was tested via correlation with the Zung Self-Rating Depression Scale, a social support questionnaire, and the risk of suicide. The authors hypothesized that the LSNS-6 would correlate with the Japanese version of the CIM in terms of support.

Self-esteem is defined as one’s attitude toward one’s own ability and strength [23]. It was measured using the Japanese version of the Rosenberg Self-Esteem scale (RSE) [24], which consists of 10 items. The RSE is the most widely used measure of self-esteem, and its psychometric validity has been confirmed in many different population samples, including patients with schizophrenia. Responses to the scale are assessed using a 5-point Likert scale. The total possible score ranges from 10.0 to 50.0, with higher scores indicating greater self-esteem. The scale’s reliability has been established with a Cronbach’s alpha of 0.81, and its validity was tested via correlation with the Brief Core Schema Scale and the Automatic Thoughts Questionnaire-Revised. McColl et al. [15] reported that participation in activities or self-determination is related to one’s perception of one’s own ability or strength. Based on the original study, we considered that the RSE would relate to the Japanese version of the CIM in terms of independent living and occupation.

Loneliness was determined using the Japanese version of the UCLA Loneliness Scale Version 3 (UCLALS) [25]. The UCLALS consists of 20 items and includes questions such as “How often do you feel left out?” “How often do you feel the lack of companionship?” and “How often do you feel isolated from others?” Each item is answered on a 4-point Likert scale. The total UCLALS score ranges from 20.0 to 80.0, with higher scores indicating higher degrees of loneliness. All 20 items are highly inter-correlated in the Japanese version (Cronbach’s alpha = 0.92), and the validity of the scale was tested via its correlation with the Geriatric Depression Scale-Short Version-Japanese. Loneliness is the unpleasant experience that occurs when a person’s network of social relations is either quantitatively or qualitatively deficient in some important ways [26]. It was hypothesized that UCLALS would correlate with the Japanese version of the CIM in terms of assimilation and support.

### Statistical analysis

Descriptive statistics were used to describe the participants’ demographic characteristics. Several statistical tests were conducted to examine the psychometric properties of the Japanese version of the CIM.

Item analysis included assessing the percentage of missing values, skewness, kurtosis, good-poor (GP) analysis, and the item-total (IT) correlation analysis. These analyses were conducted to show the normality of the variables for the application of parametric analysis and to confirm that the items were appropriate. In the GP analysis, the total points were selected, an average for each separate item was calculated, and the top 25% was classified as the “good” group while the bottom 25% was classified as the “poor” group.

Exploratory factor analysis (EFA) using maximum-likelihood estimation was conducted to determine the number of items and the nature of the underlying factors in the Japanese version of the CIM. Factor loadings equal to or greater than 0.4 were considered acceptable [27].

Internal consistency was tested using Cronbach’s alpha coefficient to determine the reliability of the instrument. A Cronbach’s alpha coefficient greater than 0.70 was considered acceptable [28].

The construct validity was examined using confirmatory factor analysis (CFA) to assess the model fitness. Various fit indices were used to assess the fit of the model to the data: the goodness of fit index (GFI), the adjusted goodness of fit index (AGFI), the comparative fit index (CFI), and the root mean square error of approximation (RMSEA). GFI, AGFI, and the CFI values greater than 0.90 were considered acceptable [29], and RMSEA values ranging from 0.08 to 0.10 were considered indicative of a mediocre fit [30].

Concurrent validity was assessed by estimating Pearson’s correlation coefficients between the Japanese version of the CIM and external criteria, including demographic characteristics and the LSNS-6, RSE, and UCLALS scores. Student’s *t* test and analysis of variance (ANOVA) were conducted to assess the relationship between demographic characteristics. If the distribution of variance showed homoscedasticity according to the Levene test, Tukey’s test was performed for multiple comparisons. All analyses were conducted using IBM^®^ SPSS for Windows version 22.0 and Amos version 20.0. Statistical significance was set at *p* < 0.05.

### Ethical approval

The questionnaires were anonymous; therefore, the private information of each participant was not identified. All the questionnaires were completed by the participants themselves and then returned directly to the researchers via mail. This study was reviewed and approved by the Institutional Review Board of the Medical Department of the Yokohama City University (Approval Number: A140724017).

## Results

### Characteristics of the study participants

The study received 402 responses (response rate: 71.8%) from individuals with any psychiatric disorder. The number of responses specifically from people with schizophrenia was 291, though 28 responses were excluded due to missing data. Thus, 263 responses were analyzed (effective response rate: 65.7%). Table 1 shows the demographic characteristics of the participants. The mean age of the participants was 46.2 (SD 9.6) years, and 182 participants (69.2%) were male. One hundred sixty-seven participants (63.5%) lived with their family, and 67 (25.5%) lived alone. Most of the participants (94.7%) were not married. One hundred sixteen participants (44.1%) had graduated from high school. Seventy-nine participants (30.0%) were working, and 153 participants (58.1%) indicated that they had insufficient or slightly insufficient budgets. Two hundred fifteen participants (82.2%) had experienced psychiatric hospitalization; of these, 57 of (21.7%) had experienced one to five cumulative years of psychiatric hospitalization (Table 1).

**Table 1:** Characteristics of study participants (n = 263)

	n	%
Demographic characteristics		
Age (years) (mean ± SD)	46.2 ± 9.6	
Sex		
Male	182	69.2
Female	81	30.8
Household membership		
Family members	167	63.5
Live-alone	67	25.5
Marital status		
Married	14	5.3
Education status		
Junior high school	43	16.3
High school	116	44.1
College or university	56	21.3
Employment status		
Working	79	30.0
Budget		
Sufficient	110	41.9
Insufficient	153	58.1
Cumulative years of psychiatric hospitalization (n = 215)		
<3 months	36	13.7
3–6 months	35	13.3
6 months–1 year	45	17.1
1–5 years	57	21.7
>5 years	42	15.9
Scores of the measures		
The Japanese version of the CIM (mean ± SD)	35.6 ± 7.7	
LSNS-6 (mean ± SD)	10.2 ± 5.8	
Social isolation (less than 12 points)	151	57.4
RSE (mean ± SD)	30.6 ± 7.8	
UCLALS (mean ± SD)	45.5 ± 11.2	

### Item analysis of the Japanese version of the CIM

The mean score on the Japanese version of the CIM was 35.6 (SD 7.7). The percentage of missing values ranged from 1.4 to 1.7%. The skewness and kurtosis for all items were within acceptable limits for parametric analysis. The GP analysis found that the “good” group scored significantly higher than the “poor” group did for all items. The IT correlation analysis ranged from 0.61 to 0.77 (Table 2). EFA was performed to determine whether the factor loading matrix possessed a unidimensional structure, and the number of factors retained was determined with a scree plot. The loading of all 10 items in the unidimensional structure exceeded the recommended value of 0.40 and ranged from 0.53 to 0.77 (Table 3), with 46.2% of the total variance explained.

**Table 2:** Item analysis of the Japanese version of the CIM

Item	Mean	SD	Median	Mode	Skewness	Kurtosis	Missing value		IT correlation
							n	(%)	
1	3.6	1.1	4.0	4.0	−0.58	−0.03	2	0.7	0.68
2	3.2	1.0	3.0	3.0	−0.37	−0.05	3	1.0	0.62
3	4.1	1.1	4.0	5.0	−1.10	0.87	5	1.7	0.65
4	3.3	1.1	3.0	3.0	−0.51	−0.12	3	1.0	0.77
5	3.4	1.2	4.0	4.0	−0.53	−0.54	4	1.4	0.65
6	3.9	1.2	4.0	5.0	−0.99	0.22	3	1.0	0.61
7	3.4	1.3	4.0	4.0	−0.59	−0.60	3	1.0	0.66
8	3.7	1.2	4.0	4.0	−0.82	0.05	3	1.0	0.65
9	3.4	1.2	4.0	4.0	−0.67	−0.21	3	1.0	0.73
10	3.6	1.1	4.0	4.0	−0.77	0.03	4	1.4	0.75
Total	35.6	7.7	36.0	39.0	−0.65	0.46	5	1.7

**Table 3:** Exploratory factor analysis of the Japanese version of the CIM (n = 263)

Item		Factor loading	Communality
4	I feel that I am accepted in this community	0.77	0.59
10	I have something to do in this community during the main part of my day that is useful and productive	0.71	0.51
9	There are things that I can do in this community for fun in my free time	0.69	0.47
1	I feel like a part of this community, like I belong here	0.66	0.43
3	I know the rules in this community, and I can fit in with them	0.61	0.37
5	I can be independent in this community	0.60	0.36
7	There are people to whom I feel close in this community	0.60	0.35
8	I know a number of people in this community well enough to say hello and have them say hello back	0.60	0.35
2	I know my way around this community	0.57	0.32
6	I like where I’m living now	0.53	0.29

Maximum-likelihood estimation

### Reliability and validity of the Japanese version of the CIM

**Fig. 1:**
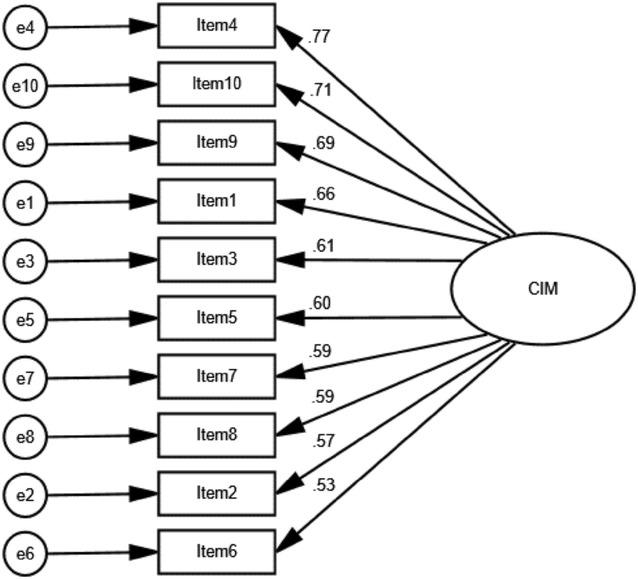
The results obtained from the confirmatory factor analysis of the Japanese version of the CIM

Cronbach’s alpha for the Japanese version of the CIM was 0.87, which indicated excellent internal consistency. CFA was performed, and the following fit indices were found: GFI = 0.924, AGFI = 0.881, CFI = 0.925, and RMSEA = 0.085 (Fig. 1). The model fit the study population exactly. These results showed that the Japanese version of the CIM was as unidimensional as the original scale. The final Japanese version of the CIM is provided in Additional file 1.

Spearman’s correlation coefficient measures relationships between external criteria and the Japanese version of the CIM. In terms of concurrent validity, significant relationships were found between the Japanese version of the CIM and the following criteria: the total score for the LSNS-6 (*r* = 0.43, *p* < 0.001) and the scores for each subscale; the RSE (*r* = 0.42, *p* < 0.001); and the UCLALS (*r* = −0.57, *p* < 0.001). Moreover, there were significant differences in the scores for the Japanese version of the CIM according to employment status (*p* < 0.05), budget (*p* < 0.05), and social isolation (*p* < 0.001) (Additional file 2). These results showed that the scores for the Japanese version of the CIM were significantly higher among participants who were employed, had sufficient budgets and were not socially isolated.

## Discussion

The results of the present study indicate that the Japanese version of the CIM is a reliable and valid instrument for evaluating the level of community integration among people with schizophrenia in Japan. To the best of our knowledge, this is the first study to develop a reliable and valid instrument for measuring the level of community integration among people with schizophrenia in the Japanese context.

The statistical evaluation of the Japanese version of the CIM was adequate, and the distribution, including skewness and kurtosis, was appropriate for the use of the parametric method. Reliability was evaluated according to internal consistency, which was sufficient, as indicated by a Cronbach’s alpha of 0.87. Both EFA and CFA were performed, and the results indicated that the one-factor structure was appropriate.

Community integration was significantly and positively correlated with social networks—the quantitative and qualitative information regarding support from one’s family and friends—and thus the concurrent validity was established. Regarding the function of social networks [31], it has been shown that individuals with a small social network tend to lack access to resources for social support. Previous studies have shown that the amount of social support has a significant positive correlation with a higher level of community integration [13]. Moreover, the present study found that the level of community integration among participants who were working and were not socially isolated was significantly higher than the level among those who were not working and were socially isolated. The level of community integration was also significantly related to loneliness and self-esteem. These results are consistent with the components of community integration. Therefore, health practitioners should work to enhance not only the skills or abilities of individuals with schizophrenia but also the community environment surrounding them.

## Conclusions

Community integration is a crucial goal for both the present and the future among people with schizophrenia. However, few studies have investigated the community integration of people with schizophrenia. The adaptation and evaluation of the Japanese version of the CIM could be applied when designing intervention programs and psychiatric care systems to promoting QOL for community-dwelling people with schizophrenia and their communities in Japan.

## Additional files

**Additional file 1: Appendix 1.** The Japanese version of the community integration measure.

**Additional file 2: Appendix 2.** CIM score difference between demographic characteristics and social isolation.

## Abbreviations

AGFI: adjusted goodness-of-fit index
ANOVA: analysis of variance
CFA: confirmatory factor analysis
CFI: comparative fit index
CIM: Community Integration Measure
EFA: exploratory factor analysis
GFI: goodness-of-fit index
GP: good-poor
IT: item-total
LSNS-6: abbreviated version of the Lubben Social Network Scale
RMSEA: root mean square error of approximation
RSE: Rosenberg Self-Esteem Scale
SD: standard deviation
UCLALS: UCLA Loneliness Scale
