# Supplementary material for: Reliability and validity of the Japanese version of the Community Integration Measure for community-dwelling people with schizophrenia
Source: Int J Ment Health Syst. 2017 Apr 17;11:29. doi: 10.1186/s13033-017-0138-2 (PMC5393028; doi:10.1186/s13033-017-0138-2)
Supplement: Supplementary file 1 — Additional file 1: Appendix 1. The Japanese version of the community integration measure. [file 13033_2017_138_MOESM1_ESM.pdf]

Appendix 1

The Japanese version of the Community Integration Measure

（日本語版 Community Integration Measure ）

以下について「いつもそうである」から「いつもそうではない」の中で、
もっともあてはまるもの1つに〇をつけてください。

1）　私は、この地域の一員であると感じる

いつもそうである　時々そうである　どちらともいえない　時々そうではない　いつもそうではない

2）　私は、この地域をよくわかっている

いつもそうである　時々そうである　どちらともいえない　時々そうではない　いつもそうではない

3）　私は、住んでいるところの生活ルールを守りながら暮らしている

いつもそうである　時々そうである　どちらともいえない　時々そうではない　いつもそうではない

4）　私は、この地域の人たちに受け入れられているように感じる

いつもそうである　時々そうである　どちらともいえない　時々そうではない　いつもそうではない

5）　私は、この地域で自立した生活ができる

いつもそうである　時々そうである　どちらともいえない　時々そうではない　いつもそうではない

6）　私は、今、住んでいるところが気に入っている

いつもそうである　時々そうである　どちらともいえない　時々そうではない　いつもそうではない

7）　この地域には、私にとって親しい人がいる

いつもそうである　時々そうである　どちらともいえない　時々そうではない　いつもそうではない

8）　この地域の人たちと私は、よくあいさつを交わす

いつもそうである　時々そうである　どちらともいえない　時々そうではない　いつもそうではない

9）　私には、この地域で楽しんでしていることがある

いつもそうである　時々そうである　どちらともいえない　時々そうではない　いつもそうではない

10）私は、この地域で充実した日課を送っている

いつもそうである　時々そうである　どちらともいえない　時々そうではない　いつもそうではない

点数化

5 点、いつもそうである；4 点、時々そうである；3 点、どちらともいえない；2 点、時々そうではない；

1 点、いつもそうではない

合計　10－50 点
