# Supplementary material for: Reliability and validity of the Japanese version of the Community Integration Measure for community-dwelling people with schizophrenia
Source: Int J Ment Health Syst. 2017 Apr 17;11:29. doi: 10.1186/s13033-017-0138-2 (PMC5393028; doi:10.1186/s13033-017-0138-2)
Supplement: Supplementary file 2 — Additional file 2: Appendix 2. CIM score difference between demographic characteristics and social isolation. [file 13033_2017_138_MOESM2_ESM.pdf]

Appendix2

Table 4

CIM score difference between demographic characteristics and social isolation (n = 263)

		Japanese ver. CIM Mean ± SD			p-value	
Sex	Male	35.9	±	8.0	ns	
	Female	35.0	±	7.1		
Household membership	Family member	35.6	±	7.1	ns	
	Live-alone	35.6	±	8.2		
Marital status	Married	35.4	±	7.8	0.097	†
	Not married	38.9	±	5.7		
Employment status	Working	37.7	±	7.1	0.030	*
	Not working	34.7	±	7.8		
Budget	Sufficient	39.0	±	8.4	0.013	*
	Slightly sufficient	35.7	±	7.6		
	Slightly insufficient	35.6	±	6.8		
	Insufficient	33.5	±	8.7		
Social isolation (LSNS-6)	Socially isolated	33.3	±	8.0	0.001	***
	Not socially isolated	38.6	±	6.1		

Student's t-test, Analysis of variance, Tukey's test

*** $p < 0.001$, * $p < 0.05$, † $p < 0.1$
